# Lecciones aprendidas de la evaluación del III Plan de Prevención de Adicciones de Navarra

**DOI:** 10.23938/ASSN.1119

**Published:** 2025-04-08

**Authors:** Fernando Baigorria Feltrin, Mikele Jauregui Elso, Jose Miguel Razkin Orobengoa, Itzal Puchol Martínez

**Affiliations:** Departamento de Salud Dirección General de Salud Instituto de Salud Pública y Laboral de Navarra Pamplona España

## Sra. Editora:

Nos dirigimos a usted en calidad de miembros del equipo de trabajo de prevención de adicciones del Instituto de Salud Pública y Laboral de Navarra. Hemos seguido con interés varios artículos publicados en su revista, Anales del Sistema Sanitario de Navarra, que abordan sustancias relacionadas con nuestro trabajo, en particular, el alcohol^1^^-^^3^.

Aprovechamos esta oportunidad para compartir nuestras reflexiones sobre la evaluación del III Plan de Prevención: Drogas y Adicciones 2018-2023 (III PPDA)^4^. Además, deseamos presentar los resultados obtenidos a lo largo de este proceso, que abarcan no solo el alcohol -como se menciona en los estudios citados- sino también todas las adicciones, con sustancia y sin sustancia. Creemos que nuestras observaciones pueden contribuir al enriquecimiento del debate en esta importante área.

El III PPDA constituye la cuarta herramienta de planificación del Gobierno de Navarra para abordar la prevención de las adicciones con y sin sustancia.

**Figura 1:**
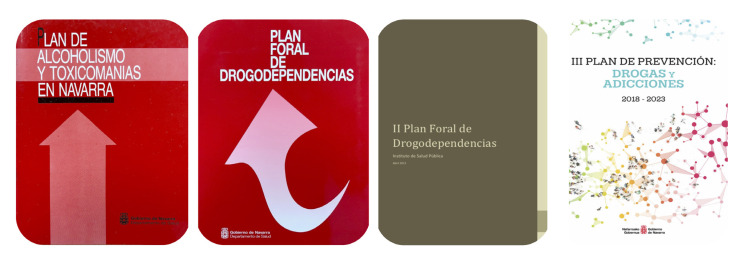
Portada de los distintos planes que han marcado las actuaciones respecto a las adicciones en Navarra desde 1986.

Históricamente el primer plan sobre adicciones en Navarra comenzó en 1986 con el Plan de Alcoholismo y Toxicomanías, que marcó el inicio de una planificación estructurada en este ámbito, y que se desarrolló en paralelo con el nacimiento del Plan Nacional sobre Drogas y enmarcado en el Plan de Salud Mental de Navarra vigente en ese momento. Aquel plan respondió a una necesidad urgente de atender a personas dependientes de opiáceos y otras sustancias, además de impulsar acciones preventivas y de rehabilitación. El siguiente fue el I Plan Foral de Drogodependencias 1993-2011(PFD) que introdujo un enfoque comunitario al priorizar la prevención y la promoción de la salud, estableciendo subprogramas específicos en entornos escolares, laborales y comunitarios. Este enfoque se consolidó con el II PFD 2012-2017^5^, que desvinculó las responsabilidades asistenciales del ámbito de la prevención, permitiendo a sus referentes centrarse en estrategias preventivas más cercanas a la ciudadanía (Fig. 1).

La novedad del III PPDA fue incorporar aprendizajes y adaptarse a los nuevos retos que planteaban los cambios sociales. Este plan se situaba en marcos de referencia, como el Plan de Salud de Navarra 2014-2020 del Departamento de Salud^6^, y el Plan de Salud Pública 2016-2020, del Instituto de Salud Pública y Laboral de Navarra (ISPLN)^7^. Asimismo, el III PPDA trabajó para alinearse con marcos nacionales e internacionales, como el Plan Nacional sobre Drogas y las directrices del Observatorio Europeo de Drogas y Toxicomanías, ahora Agencia de la Unión Europea sobre Drogas (EUDA). Este plan reforzó su foco en la prevención y promoción de la salud, con innovaciones como la inclusión del tabaco como sustancia en el III PPDA (antes enmarcado en el Plan Foral de Acción sobre el Tabaco), una mayor atención al uso de psicofármacos, y la consideración de problemáticas emergentes como las adicciones derivadas del uso de nuevas tecnologías y el aumento del juego (apuestas y videojuegos). También se consolidó el uso de cuatro estrategias en prevención de adicciones: Prevención Ambiental, Universal, Selectiva e Indicada. Todo ello se planteó desde una perspectiva de género en prevención de adicciones. Además, el III PPDA se había propuesto estar vigilante a cuestiones más complejas y sensibles como las adicciones más estigmatizadas: *chemSex*, *slamming*, consumo de drogas en prisión, policonsumo en menores, y uso no terapéutico de fármacos.

En esta carta reflexionamos sobre los aprendizajes derivados de la implementación del III PPDA y sus implicaciones para futuras estrategias. Es fundamental que las lecciones aprendidas guíen las acciones hacia un entorno más saludable y consciente para la ciudadanía de Navarra, fortaleciendo la capacidad de respuesta ante las nuevas realidades y desafíos en el ámbito de las adicciones.

## LA EVALUACIÓN DEL III PLAN DE PREVENCIÓN DE ADICCIONES DE NAVARRA

El objetivo principal de la evaluación del III PPDA^8^ fue analizar los resultados obtenidos durante su implementación, con el fin de identificar fortalezas, debilidades y áreas de mejora; durante la evaluación ISPLN contó con el apoyo de la consultoría social *Spora Sinergies*. Esta evaluación ha sido fundamental para establecer las bases del IV PPDA 2025-2030, garantizando que las futuras estrategias se alineen con las necesidades actuales de la ciudadanía y los cambios en los patrones de consumo en Navarra.

**Figura 2:**
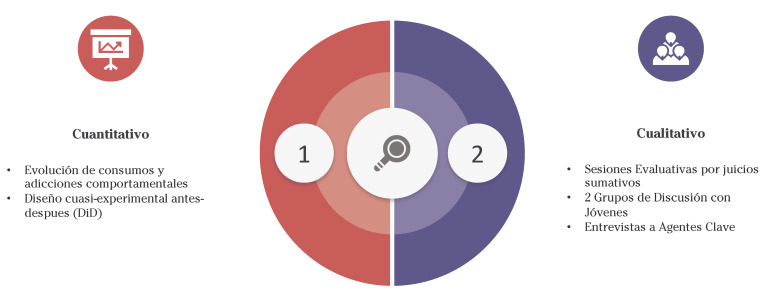
Esquema de la metodología utilizada en la evaluación del *III Plan de Prevención: Drogas y Adicciones 2018-2023*.

En el diseño se utilizó una metodología mixta. El componente cuantitativo incluyó un análisis descriptivo de la evolución sobre el consumo de sustancias y adicciones comportamentales, tanto a nivel nacional como de Navarra y se aplicó un diseño cuasi-experimental antes-después, comparando los resultados con un grupo control mediante la técnica de diferencias en diferencias, lo que permitió estimar el efecto causal del plan. Por otro lado, el componente cualitativo se centró en la recopilación de percepciones y experiencias, se llevaron a cabo dos sesiones evaluativas mediante juicios sumativos, así como dos grupos de discusión con jóvenes, y entrevistas en profundidad a agentes clave involucrados en la dirección e implementación del PPDA. Además, se promovió la participación ciudadana a través de la plataforma de Gobierno Abierto, creando un espacio donde la comunidad pudiera contribuir con sus opiniones y sugerencias (Fig. 2).

## REFLEXIONES SOBRE LA IMPLEMENTACIÓN DEL III PLAN DE PREVENCIÓN DE ADICCIONES DE NAVARRA

La implementación del III PPDA en Navarra refleja un esfuerzo por integrar la promoción de la salud y la prevención de adicciones en el tejido social de Navarra. Este proceso, liderado por la Sección de Promoción de Salud y Salud en todas las Políticas del ISPLN, ha permitido avances significativos, pero también ha enfrentado desafíos que comprometieron su impacto y en los que hay que seguir trabajando.

### - Coordinación Interdepartamental

El abordaje integral de las adicciones requiere una colaboración efectiva entre diferentes departamentos y niveles administrativos. Aunque se ha logrado avances, la coordinación entre los Departamentos con competencias en materia de Salud, Derechos Sociales, Educación, Juventud, Interior y Justicia necesita fortalecerse para garantizar políticas más efectivas. En el ámbito sanitario, la relación entre las Gerencias de Salud Mental, Atención Primaria y el ISPLN resulta crucial para asegurar la continuidad entre prevención y tratamiento, evitando intervenciones fragmentadas. La necesidad de una coordinación sólida no solo responde a la complejidad del fenómeno de las adicciones, sino también a optimizar los recursos disponibles y evitar duplicidades. Este desafío interpela a la gobernanza del sistema, que debe garantizar sinergias entre los diferentes actores involucrados.

### - Transformaciones en la Gobernanza Local

El ISPLN ha desempeñado históricamente un papel en el apoyo técnico y financiero a las entidades locales mediante subvenciones en concurrencia competitiva destinadas a proyectos de promoción de salud y prevención de adicciones. Sin embargo, una de las medidas más destacadas del III PPDA fue el traspaso de fondos destinados al cuerpo técnico de las entidades locales al Departamento de Derechos Sociales. Esta medida ha tenido una doble vertiente, por un lado, permitió en parte una estabilización del personal en los servicios sociales de base, pero también ocasionó un efecto secundario relevante: la pérdida del papel de seguimiento del ISPLN del cuerpo técnico de entidades locales en sus acciones preventivas y, como consecuencia, una falta de liderazgo en estas actuaciones. Esto ha derivado en desigualdades en la ejecución de los programas y ha acentuado disparidades territoriales, especialmente en el noreste de Navarra. Además, los profesionales de los servicios sociales de base de nueva incorporación han percibido estas tareas como una carga adicional a sus funciones habituales.

### - Trabajo en Red: Hacia una Intervención Cohesionada

El trabajo en red ha sido uno de los enfoques más valorados en la implementación del III PPDA, se ha resaltado el rol facilitador del ISPLN y su trabajo para la implantación territorial de las políticas de prevención. Sin embargo, también se resalta una falta de un liderazgo activo que ha limitado la generación de espacios de intercambio profesional y de coordinación entre los actores implicados. El crear plataformas de colaboración que permitan compartir buenas prácticas y diseñar estrategias más efectivas podrían no solo fortalecer la cohesión de las intervenciones, sino también reducir las disparidades territoriales detectadas.

### - Financiación: Un Reto Estructural

La sostenibilidad financiera era uno de los principales desafíos del III PPDA. A pesar del apoyo que suponen las subvenciones, la dependencia de recursos externos dificulta la continuidad de los proyectos a largo plazo. Es crucial desarrollar estrategias que promuevan la autonomía financiera, permitiendo que los proyectos sean sostenibles independientemente del apoyo externo.

### - Abordajes más sensibles y complejos

El III PPDA no ha logrado abordar la prevención de las adicciones profundamente marcadas por estigma social, previamente mencionadas, que siguen siendo materia pendiente en la actualidad. En cuanto al género, se ha trabajado en un informe que está pendiente de publicación sobre género y adicciones; sin embargo, aún es necesario profundizar en el análisis y en el abordaje de las actuaciones desde una perspectiva de género y la agresión sexual *oportunista* asociada a estos consumos.

Los desafíos que persisten en función del ámbito de prevención de adicciones se muestran en la tabla 1.

**Tabla 1:** Desafíos que persisten en función del ámbito de prevención de adicciones

*Prevención familiar*	
☑	Necesidad de más acciones de prevención dirigidas a mejorar las habilidades parentales y la corresponsabilidad educativa de los padres-hombres
☑	Falta de adaptación de los contenidos de talleres a la diversidad cultural, funcional y de modelos familiares
☑	Concienciación a las familias sobre sus propios consumos
*Prevención educativa*	
☑	Hay predominio de prevención universal inespecífica frente a la selectiva/indicada
☑	A pesar de haber más profesorado formado en prevención de adicciones, éste no se siente preparado o percibe una falta de tiempo para abordar la prevención en las aulas, lo que abre el debate sobre cómo abordar este tema y qué figura o figuras podrían asumirla
☑	Se ha detectado la disminución del apoyo escolar, actividad extraescolar, ofrecido por parte de entidades locales y sociales en los últimos años dificultando la accesibilidad y sostenibilidad en el tiempo
*Prevención laboral*	
☑	Falta de prevención de adicciones en el ámbito laboral en el marco de los planes de empresas
☑	Desconocimiento de hábitos de consumo en ámbito laboral
*Prevención comunitaria y colectivos vulnerables*	
☑	Falta de actividades de ocio saludable y de prevención de adicciones en los espacios de ocio
☑	La desaparición de una entidad clave en la prevención nocturna y la reducción de riesgos y daños en Navarra se ha percibido como un retroceso
☑	Programas como *PASE* y otros de ámbito local como *Voy y Vengo*, *Jaibus* y asimilados, requieren mejoras
*Prevención en ámbito asistencia*	
☑	Falta de continuidad entre ISPLN y Atención Primaria
☑	Falta de colaboración intradepartamental en el Departamento de Salud
☑	Falta de estrategias de detección precoz y el asesoramiento para la reducción de consumos

## RESPECTO A LOS INDICADORES DE CONSUMOS Y ADICCIONES COMPORTAMENTALES DEL III PLAN DE PREVENCIÓN DE ADICCIONES DE NAVARRA

Globalmente, los indicadores de consumo en Navarra han seguido una evolución similar a la observada a nivel nacional, que puede atribuirse a factores estructurales y políticas públicas similares. Navarra partía con un margen de mejora mayor, lo que ha facilitado obtener mejoras en algunos consumos.

Un logro destacado ha sido el aumento en la percepción del riesgo asociado al consumo de sustancias, especialmente entre adolescentes, en quienes el riesgo percibido ha aumentado en el consumo de alcohol, hipnosedantes y cannabis, superando incluso las cifras nacionales, resaltando el trabajo de concienciación. Sin embargo, entre la población adulta se ha registrado un aumento en la percepción de accesibilidad a sustancias como cannabis y éxtasis, indicando que las personas creen que es fácil acceder a estas sustancias, lo que requiere atención en futuras estrategias debido a las implicaciones que puede tener para la salud.

Navarra ha mostrado mejores resultados que el resto del Estado en indicadores como accidentes de tráfico e ingresos hospitalarios relacionados con el consumo de sustancias. Sin embargo, la prevalencia de víctimas en accidentes mortales de tráfico asociados al consumo de psicofármacos duplica la media estatal (11,6% frente a 6,4%), subrayando la necesidad de intensificar la prevención en este ámbito.

Entre la **juventud**, las edades promedio de inicio en el consumo de sustancias no han disminuido; aunque esta contención se interpreta como un logro conseguido, sigue siendo un desafío clave para el futuro. Un hallazgo preocupante es el aumento del botellón, especialmente entre varones, mientras que en el resto del país esta práctica ha disminuido. Además, en las chicas jóvenes resalta un consumo problemático de alcohol, incrementos en el *binge drinking* y el consumo intensivo alrededor de los 14-16 años. Al inicio de este plan en 2018, Miriam Palacios López y col^1^ ya habían señalado esta situación en Navarra, poniendo de relieve la evolución de las intoxicaciones etílicas en la población pediátrica. Estos comportamientos son el reflejo de la normalización del consumo y de los comportamientos de riesgo en nuestra comunidad foral.

El consumo de cigarrillos electrónicos, especialmente en chicas, merece atención, dado que puede estar asociado a una percepción de menor riesgo en comparación con el tabaco convencional al que, en población joven, parece remplazar^9^. Aunque el consumo de cannabis ha disminuido, sigue siendo la droga ilegal más consumida, con una normalización social preocupante. También se ha detectado que el cannabis es utilizado por jóvenes de ambos sexos ante la presión social que perciben. Se han identificado consumos emergentes utilizados en contextos recreativos y percibidos como fácilmente accesibles por las personas jóvenes y algunos colectivos específicos, como el *speed*, el *popper* y los cannabinoides sintéticos^10^. Especialmente en hombres, el consumo de bebidas energéticas ha crecido más rápido en Navarra que en el resto del estado. El consumo de cocaína sigue siendo ligeramente superior en Navarra para jóvenes entre 14-18 años, pero se ha mantenido constante. El consumo de hipnosedantes está relacionado con la patologización del malestar emocional, además de mostrar una mayor prescripción en mujeres que en hombres; en el caso de las jóvenes navarras se ha detectado un aumento en el consumo. Este comportamiento contrasta con la tendencia observada entre las personas adultas donde, como refleja el informe EDADES de 2024, el consumo ha disminuido^11^. Este fenómeno plantea nuevas preguntas sobre las vías de obtención y los factores asociados a este incremento.

El uso problemático de internet ha aumentado, especialmente entre chicas quienes, a pesar de tener mayor control parental, también enfrentan mayores riesgos de proposiciones sexuales por parte de adultos. Además, la adicción a los videojuegos y el consumo de pornografía en chicos reflejan cambios en las dinámicas de ocio juvenil y socialización, con implicaciones significativas para la salud mental.

Entre la **población adulta**, el alcohol sigue siendo la sustancia más prevalente. A pesar de que se ha observado una disminución en patrones de consumo de riesgo, especialmente en hombres, los consumos habituales han aumentado entre 45-65 años; este fenómeno refleja la normalización del consumo y su uso en contextos de ocio y socialización, lo que plantea un desafío para la salud pública debido a las consecuencias en salud como las ya descritas por Josu Delfrade y col^2^ respecto a las causas de mortalidad atribuibles al alcohol.

También se han identificado patrones preocupantes, como los consumos ocultos de alcohol en mujeres. Javier Díaz-Leiva y col señalaron acertadamente en 2021 que, aunque la demanda de ayuda por parte de mujeres está creciendo hace años, la sociedad les sigue asignando un rol de cuidadoras, lo que conlleva un mayor autocontrol aparente, menor asistencia a centros y mayor consumo a escondidas^3^. El III PPDA también señala esta problemática y advierte que, en áreas rurales, el aislamiento y la falta de redes de apoyo social pueden intensificar aún más el encubrimiento de estos comportamientos, frecuentemente vinculados al malestar emocional. Esta situación resalta la necesidad de crear espacios seguros donde las personas, y en especial las mujeres, puedan buscar ayuda sin temor a ser juzgadas.

Respecto al tabaco, se han detectado grupos de especial interés -como la comunidad gitana- donde las prevalencias de consumo de tabaco son mayores. Este aumento requiere especial atención debido al impacto en salud que tiene en el tiempo, como el aumento de incidencia de cáncer de pulmón en mujeres^12^, debido a cohortes que comenzaron a fumar más tarde que los hombres.

El consumo de cannabis entre la población adulta es similar al del resto del país, pero el consumo de riesgo ha crecido de manera más pronunciada en Navarra. Este aumento en el uso problemático indica que es necesario implementar estrategias de prevención más efectivas y adaptadas a nuestra población.

## PERSPECTIVAS A FUTURO PARA EL IV PLAN DE PREVENCIÓN DE ADICCIONES DE NAVARRA

El IV PPDA (2025-2030) deberá consolidar estos aprendizajes y adaptarse a un contexto cambiante en el que emergen nuevas formas de adicción y patrones de consumo. Será clave reforzar la gobernanza, mejorar la integración de la prevención, y garantizar la equidad territorial en la implementación de programas, así como profundizar el análisis de género teniendo en cuenta las diferencias en los consumos.

El IV PPDA se propone integrar en el trabajo nuevas herramientas incluidas en la estrategia de la Unión Europea sobre Drogas 2021-2025^13^, como el Currículum de Prevención Europeo^14^, que promueve la implementación de intervenciones de prevención responsables y basadas en la evidencia científica, las normas de calidad europeas para la prevención de adicciones y las normas internacionales sobre la prevención del uso de drogas de la UNODC y de la Organización Mundial de la Salud^15^.

La prevención debe ser una tarea compartida, con el compromiso de todos los actores involucrados: administración pública, profesionales de la salud, profesionales sociosanitarios, comunidad educativa, tejido asociativo, y la propia ciudadanía.

En definitiva, la planificación y evaluación de políticas públicas en prevención de adicciones debe seguir siendo un proceso dinámico, flexible y adaptado a las necesidades de la sociedad navarra. Solo a través de un enfoque integral y sostenible será posible avanzar hacia una comunidad más saludable y resiliente ante los desafíos que plantea el fenómeno de las adicciones.

## Data Availability

Se encuentran disponibles bajo petición a la autora de correspondencia.
